# Neurodegeneration in Niemann–Pick Type C Disease: An Updated Review on Pharmacological and Non-Pharmacological Approaches to Counteract Brain and Cognitive Impairment

**DOI:** 10.3390/ijms22126600

**Published:** 2021-06-20

**Authors:** Ida Cariati, Laura Masuelli, Roberto Bei, Virginia Tancredi, Claudio Frank, Giovanna D’Arcangelo

**Affiliations:** 1 Medical-Surgical Biotechnologies and Translational Medicine, “Tor Vergata” University of Rome, Via Montpellier 1, 00133 Rome, Italy; ida.cariati@uniroma2.it; 2 Department of Clinical Sciences and Translational Medicine, “Tor Vergata” University of Rome, Via Montpellier 1, 00133 Rome, Italy; bei@med.uniroma2.it; 3 Department of Experimental Medicine, University of Rome “Sapienza”, Viale Regina Elena 324, 00161 Rome, Italy; laura.masuelli@uniroma1.it; 4 Department of Systems Medicine, “Tor Vergata” University of Rome, Via Montpellier 1, 00133 Rome, Italy; tancredi@uniroma2.it; 5 Centre of Space Bio-Medicine, “Tor Vergata” University of Rome, Via Montpellier 1, 00133 Rome, Italy; 6 UniCamillus-Saint Camillus International University of Health Sciences, Via di Sant’Alessandro 8, 00131 Rome, Italy; claudio.frank@unicamillus.org

**Keywords:** Niemann–Pick type C, neurodegeneration, cognitive decline, lipid trafficking, pharmacological treatment, physical exercise, nutritional approach

## Abstract

Niemann–Pick type C (NPC) disease is an autosomal recessive storage disorder, characterized by abnormal sequestration of unesterified cholesterol in the late endo-lysosomal system of cells. Progressive neurological deterioration and the onset of symptoms, such as ataxia, seizures, cognitive decline, and severe dementia, are pathognomonic features of the disease. In addition, different pathological similarities, including degeneration of hippocampal and cortical neurons, hyperphosphorylated tau, and neurofibrillary tangle formation, have been identified between NPC disease and other neurodegenerative pathologies. However, the underlying pathophysiological mechanisms are not yet well understood, and even a real cure to counteract neurodegeneration has not been identified. Therefore, the combination of current pharmacological therapies, represented by miglustat and cyclodextrin, and non-pharmacological approaches, such as physical exercise and appropriate diet, could represent a strategy to improve the quality of life of NPC patients. Based on this evidence, in our review we focused on the neurodegenerative aspects of NPC disease, summarizing the current knowledge on the molecular and biochemical mechanisms responsible for cognitive impairment, and suggesting physical exercise and nutritional treatments as additional non-pharmacologic approaches to reduce the progression and neurodegenerative course of NPC disease.

## 1. Introduction

Niemann–Pick type C (NPC) disease is a rare, autosomal recessive neurovisceral disorder, caused in most cases by mutations in the *NPC1* gene (95%), and only rarely in the *NPC2* gene (5%) [1].

Lipid accumulation in the lysosomes and late endosomes is probably the crucial event in disease pathogenesis, although the underlying mechanisms are not fully understood [2]. Cholesterol has been widely recognized as the major storage lipid; however, other sphingolipid species might also be involved in the NPC pathogenesis [3,4]. Among these, sphingomyelin and glycosphingolipids have been characterized and well documented in both animal models and patients with NPC [5,6,7].

**Figure 1:**
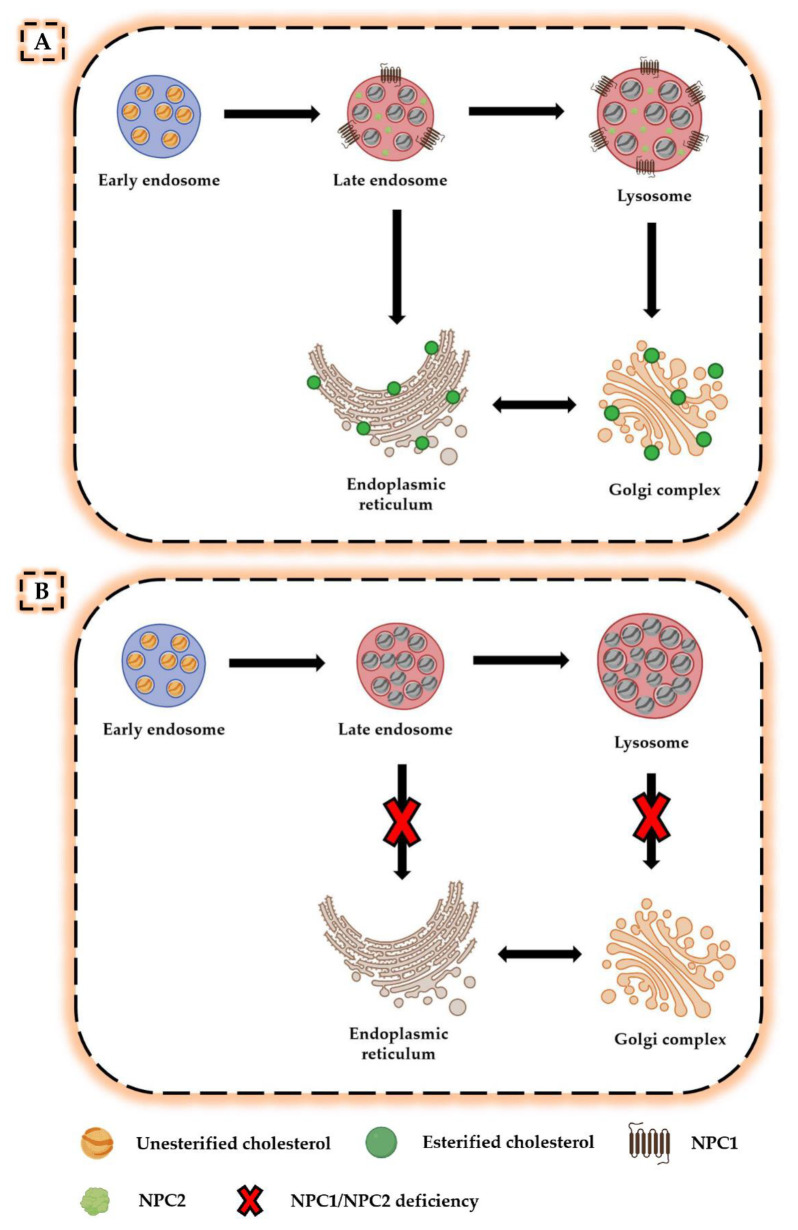
Altered cholesterol trafficking in Niemann–Pick disease type C. (**A**) In normal conditions, lipoprotein cholesterol particles bind to cell surface receptors and are internalized in the late endosome/lysosome system. In the presence of NPC1 and NPC2 proteins, unesterified cholesterol is transported from the late endosomal/lysosomal system to the Golgi complex and endoplasmic reticulum, where it is processed and used for other reactions. (**B**) In the absence of NPC1 or NPC2, unesterified cholesterol accumulates in the late endosomal/lysosomal system, resulting in deficiencies in the intracellular compartments for which it was intended.

A further advance in understanding the NPC pathogenesis was the isolation of the two genes *NPC1* and *NPC2* and the subsequent elucidation of the role played by NPC proteins in cholesterol transport. Indeed, neurons are known to obtain cholesterol through endogenous synthesis or uptake of lipoprotein cholesterol particles produced and released within the central nervous system (CNS) [8]. Following internalization of these particles by target cells, unesterified cholesterol is transported from the endosomal/lysosomal system to the Golgi complex and endoplasmic reticulum, where it is processed and used as a substrate for further reactions [9] (Figure 1A). Brown and Goldstein suggested that the NPC1 and NPC2 proteins perform a combined activity during this process, as NPC2 binds unesterified cholesterol and transfers it to the N-terminal domain of membrane-associated NPC1, thereby allowing its transport out of the late endosome/lysosome compartment [10,11]. However, in the absence of NPC1 and NPC2, lipoprotein cholesterol particles remain trapped in late endosome/lysosome system, greatly reducing cholesterol levels in the Golgi complex and endoplasmic reticulum and causing deleterious effects on all those processes that depend on proper membrane composition [12] (Figure 1B).

The idea that NPC1 and NPC2 proteins act together is now generally accepted, as confirmed by subsequent studies in model systems and computationally [2,13,14]. It has also been proposed that NPC1 transfers cholesterol from the N-terminal domain to a sterol-sensing domain (SSD); however, the mechanism of this transfer is still unknown, in part because of the highly variable results agreeing only that NPC1 possesses a cholesterol-binding site aligned with the luminal leaflet of the lysosomal limiting membrane [15,16,17,18]. Mutations in the SSD domain or in the entire NPC1 protein can cause disease [19]. To understand how loss of NPC1 function leads to neurodegeneration typical of NPC disease, Reddy et al. using cDNA microarrays analyzed the genome-wide expression patterns of human fibroblasts homozygous for the I1061T NPC1 mutation, which is the most described and is characterized by a severe defect in intracellular processing of low-density lipoprotein (LDL)-derived cholesterol [20]. Homozygous carriers of the I1061T mutation manifest a relatively mild neurological form of the disease, with onset at a young age and homogeneous clinical symptoms. NPC1 fibroblasts showed highly significant differences from control cells, with an inappropriate homeostatic response to intracellular cholesterol accumulation. Indeed, it is known that LDL receptor expression is not down-regulated in NPC cells, so LDL uptake continues to occur despite increased cellular free cholesterol content [12,21]. Microarray analysis confirmed this dysregulation, as NPC fibroblasts homozygous for the I1061T mutation showed approximately 1.5-fold higher LDL receptor gene expression than normal fibroblasts [20]. In addition, the authors observed increased expression of oxysterol binding protein like 3 (OSBPL3), oxysterol binding protein like 6 (OSBPL6), and oxysterol binding protein like 8 (OSBPL8), which belong to the family of oxysterol-binding proteins involved in the non-vesicular cholesterol transfer between the endoplasmic reticulum and the plasma membrane [22]. *OSBPL3* and *OSBPL6* have been shown to contain sequences targeting for the endoplasmic reticulum and plasma membrane, and the subsequent overexpression of oxysterol binding protein 2 (OSBP2) promotes cholesterol synthesis and is responsible for LDL receptor upregulation, probably through enhancing endoplasmic reticulum cholesterol efflux [22]. Finally, NPC fibroblasts showed a gene expression profile indicative of oxidative stress, suggesting that all these changes may contribute to the pathophysiology of NPC disease [20]. However, among various unresolved questions, it remains to be clarified whether NPC1 protein is sufficient to complete cholesterol export or whether other entities are also involved.

Using a pharmacologically induced model (U18666a, a sterol molecule that potentially interferes with NPC1 protein function) of NPC disease in macrophages, Lloyd-Evans and colleagues studied the sequence of pathological events that occur in cells following NPC1 protein inactivation [23]. The authors found that NPC1 inactivation is followed by sphingosine accumulation and an approximately 75% reduction in lysosomal calcium levels [24]. The alteration in calcium homeostasis was attributed to sphingosine storage, as treatment of NPC1 cells with myriocin, which inhibits sphingolipid biosynthesis, normalized lysosomal calcium levels [24]. Notably, exogenous administration of sphingosine induced its rapid accumulation in lysosomes, confirming the role of sphingosines in rapidly translocating across membranes and its subsequent entrapment due to protonation [23,25]. These results suggest that the reduction in lysosomal calcium content and subsequent reduced calcium release from lysosomes is directly responsible for the endocytosis defects widely observed in NPC1 cells [24].

NPC disease has several nonspecific visceral, neurologic, and psychiatric clinical features that can arise at different stages of disease and progress at different rates [26]. Clinical symptoms are heterogeneous, with an age of onset ranging from the perinatal period to adulthood. The lifespan of patients also varies from a few days to over 60 years of age, although most cases die between 10 and 25 years of age [27,28,29]. Except for the few patients who die at birth or in the first 6 months of life from hepatic or respiratory failure, all will generally develop a progressive and fatal neurological disease, characterized by cerebellar ataxia, dysarthria, and dysphagia [30].

The disease severity and its onset might depend on the degree of functional disruption of cholesterol trafficking [31]. Particularly, an earlier onset of disease has been suggested in cases in which the degree of functional disruption of cholesterol trafficking is more severe, whereas a later onset and slower course might be associated with a less severe degree of functional disruption of cholesterol trafficking [29]. The disease severity is also related to a genetic component. In this regard, Wassif et al. analyzing NPC1 variants identified the p.H215R variant in almost one third of NPC1 alleles [32]. Furthermore, they demonstrated that two relatively common NPC1 variants with a combined carrier frequency approaching 0.8% may contribute, in compound heterozygous state, to a late-onset NPC1 phenotype for which the phenotypic spectrum and clinical significance have yet to be defined [32]. According to the authors, this late-onset NPC1 phenotype may represent a milder manifestation of NPC1 deficiency with predominantly visceral manifestations, although further studies are needed to define the degree to which this phenotype is associated with high frequency NPC1 alleles. Thus, the true prevalence of NPC disease is difficult to assess, due to insufficient clinical awareness, incomplete ascertainment of atypical phenotypes, as well as limitations of current diagnostic tests [32].

Finally, despite much research over the years, the treatment of NPC disease remains very limited, and nothing is known about the use of possible non-pharmacological treatments that could help in preventing and/or delaying neurodegeneration in NPC patients. Therefore, the aim of our review was to (i) summarize the current knowledge about the molecular and biochemical mechanisms responsible for cognitive impairment, and (ii) suggest how physical activity and appropriate diet may represent additional strategies to existing pharmacological approaches to reduce the progression and neurodegenerative course of NPC disease.

## 2. Neurodegeneration in NPC Disease

**Figure 2:**
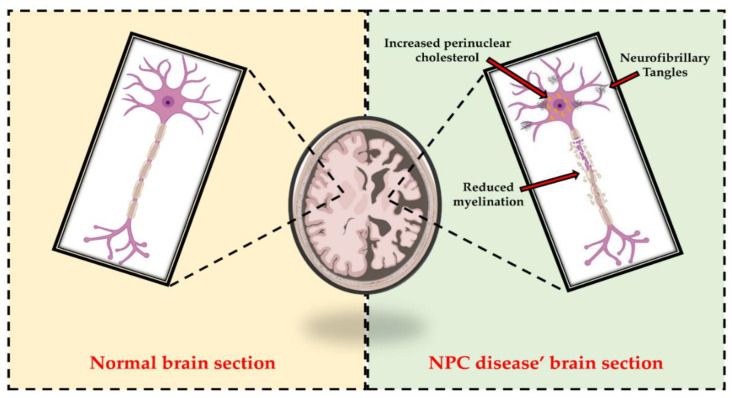
Representative comparison between normal brain section and NPC disease’ brain section. As highlighted in the right panel, alterations in cholesterol homeostasis are associated with disruption of brain function and the onset of neurodegeneration. In fact, increased perinuclear cholesterol, reduced myelination, and neurofibrillary tangles (NFT) accumulation are among the hallmarks of NPC patients’ neurons.

Given the key role of cholesterol in the regulation of membrane biophysical properties and cellular functions through the modulation of several signaling pathways, alterations in cholesterol homeostasis have been associated with disruption of brain function and the onset of neurodegeneration [33] (Figure 2). In this regard, Schultz et al. reported that the neuronal loss characterizing NPC disease is related not only to the composition and morphology of synaptic vesicles, but also to endosomal organelle transport [34]. Furthermore, Malnar and colleagues explained that the characteristic ataxia of NPC patients was closely dependent on the increased vulnerability of Purkinje cells in the cerebellum to the disease [35]. *Npc1^−/−^* mice, which are used in experimental practice as animal models for NPC1 disease, also mimic most of the pathologic features of NPC patients [36,37,38]. In fact, they exhibit not only loss of neurons, but also increased levels of cholesterol in mitochondria from brain and hepatocytes, highlighting the close relationship between the two organelles in cholesterol trafficking [34,39,40].

Notably, several pathological similarities have been identified between NPC and other neurodegenerative disorders, such as Alzheimer’s disease (AD). Particularly, progressive neurodegeneration, cholesterol accumulation and subsequent late endosome/lysosome abnormalities, Tau hyperphosphorylation, neurofibrillary tangles (NFTs), and β-Amyloid (Aβ) accumulation are known to occur in both NPC and AD [33,41,42]. In fact, although amyloid plaque formation has not been observed in the brains of NPC patients, in vivo and in vitro studies have shown that the altered cholesterol trafficking observed in NPC disease may modulate β-amyloid precursor protein (APP) processing [35]. Specifically, Malnar et al. observed in *Npc1^−/−^* cells a decreased APP expression on the cell surface and increased APP processing through the β-secretase pathway, resulting in increased levels of C99 and Aβ intracellular. The authors suggested that this effect was dependent on increased cholesterol levels, since cholesterol depletion not only reversed APP expression on the cell surface but also reduced Aβ and C99 levels in *Npc1^−/−^* cells with values comparable to those observed in control cells [43]. Therefore, these results confirmed the role played by cholesterol in APP metabolism, demonstrating the existence of a correlation between cholesterol homeostasis, APP metabolism, and AD pathogenesis.

In agreement, other scientific evidence reported increased levels of Aβ, Aβ 42, and β-C-terminal fragments (β-CTF) in experimental models of NPC disease [44,45,46]. Specifically, Burns et al. showed that aberrant intracellular cholesterol transport in *Npc1* mutant mice was associated with both profoundly altered β-CTF levels, γ-secretase activity, and subcellular distribution of presenilin-1 (PS-1) and increased formation of Aβ 40 and Aβ 42 peptides [47]. Since late endosomes have been reported to act as a site for Aβ and PS-1 accumulation in NPC cells [44,45], the authors using a sucrose gradient to separate late and early endosomes observed in *Npc1* mutant mice that, while PS-1 was present in the endoplasmic reticulum, and also accumulated in organelles with similar buoyancy to the early endosomal fractions (Rab 5 positive), no PS-1 was detected in the late endosomal fractions (Rab 7 positive). Furthermore, confocal microscopy analysis showed that PS-1 and Rab 5 immunoreactivity overlapped in brain tissue of *Npc1* mutant mice but not in control mice. These findings, which agree with recent data from human NPC postmortem brains [46], highlight the role of cholesterol in amyloidogenesis, and provide information about the characterization of the *Npc1* mutant mouse model that may help identify common pathological features between AD and NPC disease [47].

Gene expression analysis conducted by Reddy et al. of fibroblasts from patients homozygous for the I1061T NPC1 mutation revealed many interesting similarities with classic neurodegenerative diseases [20]. First, a significant increase in the generation of β-CTF at the protein level in human NPC1 fibroblasts compared with normal fibroblasts was confirmed. Genes encoding for LDL receptor-related proteins (LRP1, LRP2, and LRP6) were also upregulated by 1.8-, 2.2-, and 2.7-fold, respectively, in NPC fibroblasts. In addition, both mRNA and amyloid-beta precursor binding protein 2 (APBB2), which is known to interact with the cytoplasmic domain of APP and allow its cleavage by γ-secretase [48], were significantly upregulated by approximately 2.5-fold, thus contributing to the increased generation of Aβ 42 in NPC cells [49]. Based on their findings, the authors concluded that genes associated with AD are upregulated in NPC cells [20].

Subsequently, Kagedal and colleagues measured gene and protein expression of NPC1 in three distinct regions of the human brain, observing that its expression was upregulated in both the hippocampus and frontal cortex of AD patients compared with control subjects, whereas no difference was detected in the cerebellum [50]. In addition, a stronger expression of NPC1 was observed in hippocampal neurons, as well as reduced total cholesterol levels were found in the hippocampus of AD patients compared with control individuals. Thus, in agreement with other studies, it was suggested that the increased expression of NPC1 was related to altered cholesterol homeostasis in AD [50].

Recently, microglial changes have also been considered among the primary pathological events in neurodegeneration in NPC disease. Particularly, it has been observed that NPC1 plays a key role in the formation and maintenance of CNS myelin by oligodendrocytes, and that an alteration in intraneuronal lipid transport is closely related to reduced oligodendrocyte maturation and significant white matter hypomyelination [51,52]. These findings were largely confirmed by neuroimaging and brain magnetic resonance imaging (MRI) studies, which revealed diffuse axonal and myelinated gray and white matter changes in NPC disease, as well as volumetric changes at the level of the cerebellum, hippocampus, cortex, thalamus, and caudate nuclei [53,54]. Notably, a reduction in these changes and in the progression of volume loss was observed following treatment of NPC patients with miglustat [55].

### 2.1. Molecular and Biochemical Events

Previous studies have established that neurodegeneration-associated changes in NPC disease depend on a more rapid turnover of the cholesterol pool in CNS [56,57,58].

First, it has been proposed that increased degradation and excretion of sterol across the blood–brain barrier could occur due to a functional alteration of cholesterol 24-hydroxylase (CYP46A1), which is responsible for the formation of 24(S)-hydroxycholesterol in the brain [59,60]. However, Lund et al. by mRNA hybridization studies and immunohistochemical analyses localized the enzyme to both pyramidal cells in the cortex and Purkinje cells in the cerebellum, excluding the involvement of CYP46A1 in the metabolism of extracellular cholesterol released during neurodegeneration [61]. In agreement with these results, *Npc1* mutant mice showed a significant reduction in the rate of CYP46A1 excretion simultaneously with an increase in net cholesterol excretion from the CNS [59,60].

Second, Repa and colleagues hypothesized that excessive cholesterol release during neurodegeneration could activate the liver X receptor (LXR) system, thereby increasing transcription of proteins involved in sterol degradation or transport from the CNS [62]. However, this hypothesis was refuted by Li et al., who found no change in mRNA levels in *Npc1^−/−^* mice for ATP-binding cassette transporter (ABCA1), ATP-binding cassette sub-family G member 1 (ABCG1), fast cell surface death receptor (FAS), stearoyl-CoA desaturase-1 (SCD1), stearoyl-CoA desaturase-2 (SCD2), and sterol regulatory element-binding protein 1 (SREBP1c), concluding that none of the LXR target genes were activated in the presence of neurodegeneration [58].

Finally, it was proposed that apoproteins expressed in the brain might also play a role in the transport of cholesterol from degenerating cells to the blood–brain barrier [58]. In this regard, it was observed that *Npc1^−/−^* mice showed a significant increase in mRNA levels for both apolipoprotein E (apoE) and apolipoprotein D (apoD), which is known to occur in the presence of nerve damage in the central or peripheral nervous system [58,63,64].

Taken together, altered cholesterol homeostasis could involve at least three different systems. Presumably, altered cholesterol transport from the late endosome/lysosome compartment could directly or indirectly cause apoptotic Purkinje cell death, triggering both microglia and astrocyte activation and proinflammatory proteins secretion [65]. Simultaneously, increased synthesis of apoE and apoD, which also play a role in the excessive transport of sterol to sites where it can be excreted from the CNS, may occur [58]. However, further studies will be required to better elucidate the molecular and biochemical dynamics that trigger the neurodegenerative process in NPC disease.

### 2.2. Hyperexcitability and Altered Glutamatergic Neurotransmission in NPC Disease

Although the pathophysiological basis is still poorly understood, NPC patients show hyperexcitability among their main symptoms, with consequent epileptic manifestations [66,67]. The cause of this phenomenon has been attributed to the dynamic loss of lipid rafts enriched in cholesterol and sphingolipids, which occurs because of an imbalance in lipid trafficking [68]. Indeed, cholesterol is known to be involved in the synapse’s organization and especially in the formation of lipid rafts, which are membrane microdomains that play a key role in most cellular functions [69]. In this regard, Frank and colleagues observed altered synaptic transmission and plasticity mediated by alpha-amino-3-hydroxy-5-methyl-4-isoxazole-propionic acid (AMPA), kainate (KA), and N-methyl-D-aspartate (NMDA) receptors in cholesterol-poor hippocampal neurons [70]. In fact, memory-related brain structures, such as the hippocampus, are known to be directly involved in the epileptic process, since cognitive deficits are quite common in patients with epilepsy, especially for people with temporal lobe epilepsy (TLE) [71,72].

Accordingly, hypothesizing that hyperexcitability was related to dysregulation of glutamatergic system function, we previously studied excitatory neurotransmission by basal synaptic transmission (BST) recordings in the pyramidal layer of the CA1 region of hippocampal slices from *Npc1^−/−^* mice and *Npc1^+/+^* mice [73]. Increased excitability in the hippocampus of mutant mice was observed, with significantly higher population spikes (PSs) amplitude values than those of *Npc1^+/+^* mice. To test whether the increased BST was due to enhanced glutamate release, we applied a paired pulse facilitation (PPF) protocol, consisting of a short lasting presynaptic alteration in synaptic efficacy determined by neurotransmitter release. Indeed, a significantly higher PPF ratio was detected in hippocampal slices of *Npc1^−/−^* mice compared with *Npc1^+/+^* mice, indicative of an increase in neurotransmitter release from presynaptic terminals and, therefore, of enhanced excitability [73]. Moreover, to better understand the molecular mechanisms underlying the increased BST observed in *Npc1* mutant mice, electrophysiological recordings were also performed in hippocampal slices perfused with AMPA and KA, which are considered glutamate receptor agonists. Treatment with KA induced a significant increase in PS amplitude values in hippocampal slices from *Npc1^+/+^* mice, followed by complete abrogation of the field potential; whereas treatment with AMPA resulted in a strong reduction in BST 30 min after administration. In contrast, hippocampal slices from *Npc1^−/−^* mice responded to KA treatment only with a slight reduction in BST, whereas application of AMPA did not result in alterations of field potential amplitudes [73]. Further confirmation was provided by Calcium Imaging analyses, which revealed significant differences in calcium influx during AMPA and KA treatment in the hippocampal slices of mutant mice compared with those of *Npc1^+/+^* mice. Taken together, our results suggest that an increase in glutamatergic excitatory transmission could underlie the increase in synaptic activity recorded in hippocampal slices of *Npc1^−/−^* mice, either because of reduced KA receptor activity or a failure of the mechanism of glutamate- or agonist-induced AMPA receptor internalization, suggesting a key role of altered glutamatergic neurotransmission in the genesis of epileptic activity typical of NPC disease [73].

The progressive neural loss typical of NPC disease could also be triggered by a hypoxic insult, to which the hippocampus is extremely susceptible [74]. Particularly, it has been suggested that hypoxic insults, simulated in vitro by transient oxygen/glucose deprivation (OGD), could result in LDL oxidation and lectin-like oxidized LDL receptor (LOX-1) activation, generating ischemic long-term potentiation (i-LTP). This pathological form of synaptic plasticity, in turn, would cause an increase in intracellular calcium and consequent apoptotic death of neurons [74,75].

To test the actual involvement of hypoxic insult in NPC pathogenesis, Lo Castro and colleagues recently investigated the electrophysiological response of hippocampal slices from *Npc1^−/−^* and control mice to ischemic insult, observing the development of an early i-LTP in mutant mice during OGD application [76]. In addition, LOX-1 expression was evaluated by RT-qPCR analysis, immunocytochemistry, and Western blot before and after the anoxic episode. It was observed that following OGD, LOX-1 transcript and protein expression increased in both *Npc1^−/−^* and control mice, although protein expression was delayed, probably due to kinetics of different induction; in contrast, prior to OGD, increased expression of the LOX-1 transcript and protein was detected only in mutant mice. In conclusion, the authors hypothesized the existence of a correlation between impaired cholesterol transport, caused by the NPC1 protein lack and oxidative stress, and the regulation of oxidized LDL receptor levels on the cell membrane in brain tissues, indicating oxidative stress as another probable cause of the high susceptibility of *Npc1^−/−^* mice to neurodegeneration [76].

## 3. Therapeutic Approaches Used to Counteract Cognitive Deficits

Despite the prevalence of cognitive impairment and its negative impact on functioning and quality of life, there is currently no cure for NPC disease. Research on possible disease-modifying therapies began in the 1950s and focused on studying the therapeutic effect of lipid-lowering agents, since unesterified cholesterol was originally considered the major metabolite underlying biochemical damage [77,78,79]. However, several experimental evidences showed that therapies aimed at cholesterol reduction were ineffective in counteracting cognitive impairment [80]. In this regard, Erickson et al. showed that treatment with nifedipine, a calcium channel blocker that induces cholesterol efflux, and probucol, an inhibitor cell surface cholesterol exporter ABCA1, reduced hepatic cholesterol levels in *Npc1* mutant mice but had no effect on disabling neurological symptoms [81]. Similarly, Beheregaray and colleagues investigating the effect of clofibrate, a peroxisomal proliferating agent, on intracellular cholesterol accumulation in cultured fibroblasts from NPC patients, showed that it was not helpful in treating the disease, but on the contrary contributed to increased cholesterol levels in the cells of these individuals [82]. Thus, although treatment with lipid-lowering agents caused reductions in hepatic and plasma cholesterol levels in experimental mouse models and NPC patients, it had no impact on the neurological progression of the disease [80].

### 3.1. Miglustat Treatment

For many years, neurological manifestations have been treated with palliative approaches, including antiepileptic drugs, anticholinergic drugs to relieve dystonia and tremor, and antidepressant or antipsychotic drugs for mood and psychosis disorders [26,83].

Subsequently, the research proposed miglustat, a small iminosugar molecule that inhibits the synthesis of glycosphingolipids, as the first and only targeted therapy for the treatment of NPC patients [84,85,86]. Since its initial approval in Europe in 2009, the clinical experience with miglustat in the treatment of NPC disease has increased dramatically, as documented in numerous experimental studies.

Recently, Bradbury et al. suggested a role for calcium homeostasis in the therapeutic effects of miglustat, given the discovery of reduced levels of calcium-binding proteins in cerebellar neurons [87]. Indeed, Lloyd-Evans had previously shown that miglustat could modulate intracellular calcium homeostasis through its effects on glucosylceramide levels [88]. Confirming this hypothesis, one of the initial factors in NPC pathogenesis is believed to be the impairment of calcium homeostasis related to excessive sphingosine accumulation, which inhibits lysosomal calcium uptake, causing reduced endocytic function and subsequent development of the NPC disease phenotype [23,24].

Numerous experimental evidences have demonstrated the efficacy of miglustat in delaying the progression of neurodegeneration in NPC [89,90,91]. For example, it has been reported that miglustat reduced neuronal glycosphingolipid accumulation, delayed the onset of neurological dysfunction, and prolonged survival in NPC animal models [92,93]. Improved Purkinje cell survival in cats following miglustat treatment was also observed, probably related to modulation of microglial immunophenotype and function [94].

In agreement with other studies, we recently investigated whether in vivo treatment with miglustat could counteract cognitive impairment in an NPC mouse model [95]. Specifically, we analyzed by means of in vitro extracellular recordings in hippocampal slices from *Npc1^−/−^* mice, the effects of miglustat treatment on long-term potentiation (LTP), known to be the electrophysiological paradigm of learning and memory and whose impairment correlates with the onset of cognitive deficits [96]. We observed that miglustat administration was not only able to reverse the previously reported hyperexcitability in hippocampal slices of *Npc1^−/−^* mice, but also to counteract the impairment of LTP, whose induction and maintenance phases were significantly reduced. Notably, we observed that restoration of synaptic plasticity was correlated with significant extracellular signal-regulated kinase (ERK) phosphorylation, suggesting a direct effect of miglustat on synaptic enhancement signaling pathways. Overall, our data highlight the efficacy of miglustat in the treatment of neurological deficits associated with NPC disease, although further studies are needed to better understand the exact mechanism of action of miglustat and improve its therapeutic role [95].

### 3.2. Cyclodextrin Treatment

Recent evidence has proposed hydroxypropyl-β-cyclodextrin (HPβCD) as a new line of treatment for NPC disease, as it could minimize neurological damage, reduce symptoms, and delay progression [97,98]. The therapeutic efficacy of HPβCD was discovered casually, following its use as an excipient to administer allopregnanolone in an NPC1 mouse model [99]. Indeed, neurosteroids, steroids produced in the brain, are known to influence neuronal growth and differentiation, as well as modulate neurotransmitter receptors [100,101]. Griffin et al. suggested that altered cholesterol transport might block the process of neurosteroidogenesis, thus contributing to the development of the NPC phenotype [102]. Indeed, the authors observed a lower neurosteroid content in the brains of NPC mice compared with that in control mice, as well as an age-related decrease in the ability to synthesize allopregnanolone. Surprisingly, the decrease in allopregnanolone production appeared to contribute to the NPC pathogenesis, suggesting neurosteroid treatment as a useful strategy to ameliorate disease progression [102].

However, subsequent studies demonstrated that HPβCD, rather than the neurosteroid, was the active party. Specifically, Davidson et al. suggested that cyclodextrin administration to *Npc1^−/−^* mice delayed clinical disease onset, reduced intraneuronal cholesterol levels and glycosphingolipid accumulation, as well as reduced expression of neurodegeneration markers and increased animal survival [103]. Similarly, Liu and colleagues demonstrated that a single dose of cyclodextrin administered at 7 days of age in *Npc1^−/−^* mice was able to reverse the lysosomal transport defect observed in NPC disease, reducing macrophage activation and influx into the liver and brain, significantly improving liver function and Purkinje cell survival, as well as reducing neurodegeneration and increasing lifespan [104]. In addition, intrathecal administration of the drug in a single NPC1 patient resulted in increased cholesterol redistribution in the CNS, with similar efficacy to that observed in murine and feline models [105]. Finally, Ory et al. reported that intrathecal HPβCD administered to 14 NPC patients slowed disease progression with an acceptable safety profile [99].

Despite promising results, the use of HPβCD has several limitations for the treatment of NPC disease. First, induction of the therapeutic response and subsequent cholesterol clearance requires an extremely high concentration of the drug [106]. Second, neurological symptoms can only be alleviated by intrathecal administration of the drug, as it is not capable of crossing the blood–brain barrier [107]. Finally, side effects, including hearing loss, have been observed following administration of high doses of the drug [108,109]. Because of these limitations, the optimal dose and dosing intervals for HPβCD remain to be determined, necessitating further studies aimed at understanding the mechanisms of action of this drug in the NPC pathogenesis.

## 4. Additional Non-Pharmacologic Approaches for Neurodegeneration

### 4.1. Physical Exercise

In recent years, numerous researchers have supported the importance of exercise in the prevention and treatment of neurodegenerative diseases [110,111,112]. The central aim of such research has been to understand the mechanisms by which exercise is able to reduce and delay the onset of the symptomatology of neurological disorders, to identify the best protocols for patients [113]. In this regard, it is now generally accepted that regular physical activity promotes the release of myokines and metabolites into the circulation during muscle contraction [114]. These molecules can cross the blood–brain barrier at the level of brain capillaries and influence the functions of neurons and glial cells, thus modifying neurotransmission in different brain regions [113].

More importantly, physical activity is known to influence and improve cognitive processes, such as memory and learning, in animal models through its actions on the hippocampus [115]. For example, exercise, such as running, has been shown to increase neurogenesis in the dentate gyrus, as well as improve LTP and mnemonic function [116,117,118]. Similar results have also been found in human studies, where aerobic exercise has been shown to increase hippocampal volume and reduce age-related decline in mnemonic function [119,120].

Despite numerous studies over the years, specific exercises cannot yet be prescribed to maximize their positive effects on cognitive processes. This also depends on the fact that the levels of molecules released during muscle contraction change during and after exercise. In addition, it is still unclear how brain functioning may vary with the type, intensity, and timing of exercise.

#### Effects of Physical Exercise on Synaptic Plasticity in Npc1 Model Mice

Considering the various pathological similarities between NPC disease and other neurodegenerative disorders, we evaluated for the first time whether exercise could also have beneficial effects on cognitive processes in experimental NPC models.

**Figure 3:**
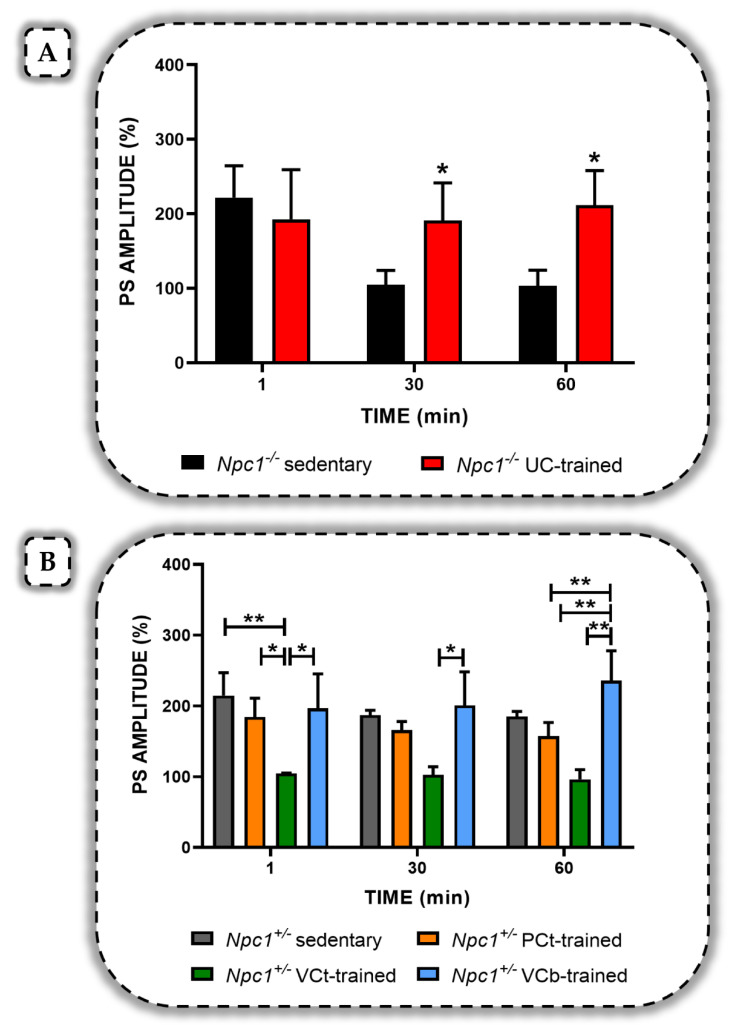
Effects of different aerobic exercises on synaptic plasticity in *Npc1* mutant mice. (**A**) The % population spikes (PS) amplitude as a function of time after tetanic stimulation is shown in *Npc1^−/−^* sedentary (black bar) and in *Npc1^−/−^* UC-trained (red bar) mice slices at minutes 1, 30, and 60. Bars in the plot are means ± SEM of values obtained from different slices. The suppression of long-term potentiation (LTP), which characterized *Npc1^−/−^* sedentary mice was completely counteracted in *Npc1^−/−^* UC-trained mice, with significantly higher PS amplitude values at minutes 30 and 60 of recording (* *p* < 0.05) [121]. (**B**) The % PS amplitude as a function of time after tetanic stimulation is shown in *Npc1^+/−^* sedentary (grey bar), in *Npc1^+/−^* PCt-trained (orange bar), in *Npc1^+/−^* VCt-trained (green bar), and in *Npc1^+/−^* VCb-trained (blue bar) mice slices at minutes 1, 30 and 60. Bars in the plot are means ± SEM of values obtained from different slices. Only the VCb protocol positively modulates synaptic plasticity, since the LTP maintenance phase was characterized by higher PS amplitude values compared with those in experimental groups (** *p* < 0.01) [122].

Previously, we showed that improvement in synaptic plasticity depended on the proposed training protocol, and that exercise exerted positive effects on cognitive processes if the workload was adequate and appropriate recovery periods were observed [96]. In line with this evidence, we recently evaluated whether exposure of *Npc1^−/−^* mice and *Npc1^+/−^* mice to a uniform continuous (UC) protocol, which is a type of short-term aerobic training administered using a Rotarod, could modulate synaptic plasticity, both qualitatively and quantitatively [121]. Each training session was characterized by a speed of 9 laps per minute (RPM) for a duration of 30 min (Table 1). Furthermore, the protocol was performed three times a week from the thirtieth to the fiftieth day of the animals’ life, for a total of three weeks. Our results showed that the UC protocol did not improve synaptic plasticity in *Npc1^+/−^* mice compared with the sedentary control group, but on the contrary, a deterioration in the LTP induction phase was evident. Surprisingly, a significant effect of training was found in *Npc1^−/−^* mice, as suppression of LTP, which was present in the sedentary control group starting at 10 min after tetanic stimulation, was completely counteracted (Figure 3). Notably, the increase in PS amplitude values observed in hippocampal slices from homozygous trained mice was similar to that found in sedentary heterozygous mice [121]. We also performed transmission electron microscopy (TEM) analysis to assess the presence of any differences in the hippocampus ultrastructure of the various experimental groups. Specifically, a recovery was found in the hippocampus of *Npc1^+/−^* trained mice compared with sedentary heterozygous mice, mainly represented by a greater number of axons. *Ncp1^−/−^* trained mice showed better tissue organization and a significant decrease in axonal vacuolization observed in contrast in homozygous sedentary mice. Interestingly, both the number and size of axon structure was comparable to that observed in the hippocampus of sedentary *Npc1^+/−^* mice. Overall, our data suggest that the short-term UC protocol, although not able to reverse the disease itself, improves LTP in the *Npc1* mutant mice [121].

Since the UC protocol was not found to be effective in *Npc1^+/−^* mice, probably because it was too weak and not demanding enough, we more recently evaluated the influence of exercise on neuroplasticity by subjecting *Npc1^+/−^* mice to more challenging training protocols over a longer period [122]. Specifically, we administered two aerobic training protocols, progressive continuous (PC) and varying continuous (VC), which differed in terms of speed and velocity changes, as described previously [123] (Table 1). The PC protocol featured a gradual rotational speed that increased from low to high intensity (10-32 RPM); whereas, the VC protocol consisted of two 8-min reverse bipyramidal series, with 2 min of active recovery between series at 10 RPM. Both protocols had a total duration of 18 min. In addition, training sessions were conducted three times per week for twelve weeks (PCt and VCt, respectively) and, in the case of the VC protocol, also twice per week (VCb) [122]. Our results showed that the PCt protocol was not efficient enough as demonstrated by impairment in the LTP induction phase, whereas the VCt protocol was even more stressful and completely inhibited synaptic plasticity. Surprisingly, the VCb protocol exerted a positive effect on LTP maintenance phase (Figure 3), since we measured higher PS amplitude values in *Npc1^+/−^* trained mice than in the control sedentary group [122].

Overall, our data confirm the efficacy of exercise on quality of life and indicate that specific aerobic training programs, such as the UC and VCb protocols, can induce short- and long-term changes in hippocampal circuits of *Npc1^−/−^* and *Npc1^+/−^* mice, positively modulating synaptic plasticity and thus improving neurodegenerative symptoms. However, although the results are promising, it should be emphasized that these are preclinical studies conducted in animal models of NPC disease and that the only treatments currently used to reduce progression and neurodegeneration in NPC patients are pharmacological.

### 4.2. Nutrition

Nutritional treatments may be considered another interventional approach to reduce symptoms and increase the lifespan, although there is very limited evidence on NPC disease and the few studies in the literature have mostly been conducted in experimental animal models.

The ketogenic diet has been used in patients suffering from inherited metabolic diseases (IMD) that present with seizure disorders [124], and has been proposed to NPC patients associated with miglustat administration to reduce seizure activity and ameliorate gastrointestinal side effects linked to the pharmacological therapy [125].

The ketosis induced through the ketogenic diet could restrict generation of reactive oxygen species and, thus, might prevent apoptosis. In this perspective, the use of antioxidants in the diet might be suggested to reduce subcellular stress in NPC patients. There is already scientific evidence indicating how the use of antioxidants in the NPC animal model can be beneficial. Vitamin E supplementation in fact delayed loss of weight, enhanced coordination and locomotor function, and increased the lifespan, improving the neurological symptoms in the *Npc1* mutant mice [126]. This result points out how the use of this vitamin in the diet could be useful for the treatment of NPC patients. Furthermore, beneficial effect of nicotinamide is also reported in counteracting cognitive impairment and enhancing survival [127].

## 5. Conclusions

NPC is a disease with many unresolved issues. First, the precise functions of NPC1 and NPC2 proteins are still largely unknown. Second, although there is growing interest in understanding the effects of NPC disease on neuronal function, little is known about alterations in the transport of cholesterol and other lipids in the CNS. Indeed, the brains of NPC patients and mouse models are characterized by lipid accumulation, dendritic, and axonal dystrophy, as well as demyelination and neuronal cell loss in various cerebral regions. Changes in cholesterol content could affect the functional properties of ion channels and membrane receptors, causing the typical clinical manifestations seen in both NPC patients and animal models of the disease. However, the mechanism underlying cholesterol accumulation, as well as how it causes neurodegeneration, has yet to be elucidated.

**Figure 4:**
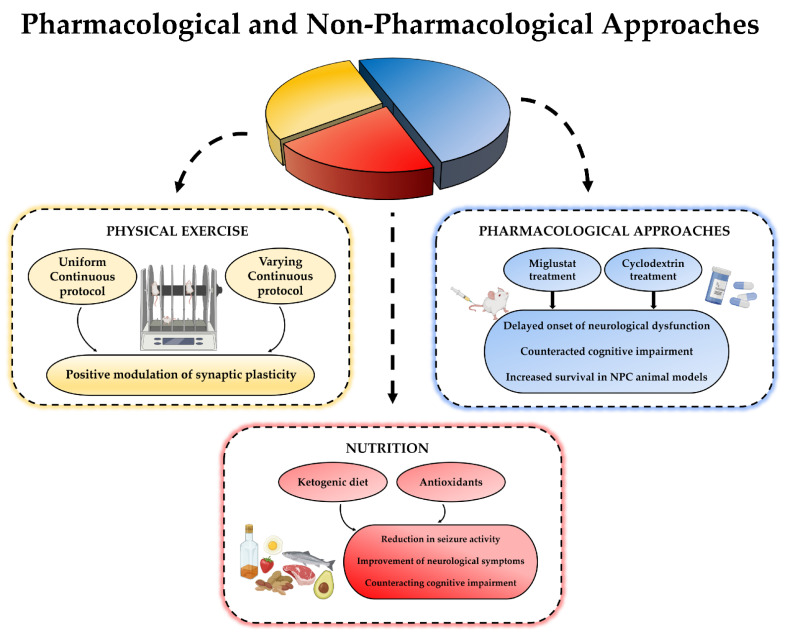
A schematic image of the combination between pharmacologic and non-pharmacologic approaches for NPC disease.

Although NPC disease is predominantly characterized by cognitive impairment, there is currently no real cure and over the years, therapies aimed at reducing cholesterol levels have been ineffective in counteracting neurodegeneration. The only pharmacological treatments used include the administration of miglustat, known to delay the progression of neural disorders, and cyclodextrin, recently discovered to minimize neurological damage and reduce clinical symptoms. For this, new non-pharmacological and assistive approaches may be useful. For example, regular physical activity, when combined with current drug therapy, could be effective in reducing the progression and course of NPC disease. Particularly, specific short- and long-term training protocols, appropriately designed in terms of workload and recovery periods, could induce adaptive changes in cognition and brain plasticity. Similarly, well-designed nutritional approaches, based on a ketogenic diet or the use of antioxidants, could reduce symptoms and increase lifespan when associated to drug therapy. Thus, the combination of pharmacological and non-pharmacological therapies could be critical in improving the quality of life of NPC patients (Figure 4).

## Figures and Tables

**Table 1:** A schematic description of the different aerobic exercise protocols used to train *Npc1* mutant mice [121,122].

	UC Protocol	PC Protocol	VC Protocol
**Main features**	Single session training at 9 RPM, without speed changes	Incremental speed changes with gradually increasing exercise intensity. Intensity increases in 2 RPM intervals from 10 to 32 RPM, with 12 speed changes	Two series of 8-min bi-pyramidal inversion exercise, with a 2-min active recovery at 10 RPM between series
**Training session duration**	30 min	18 min	18 min
**Weekly frequency**	3 times a week	3 times a week	3 times (VCt) or 2 times (VCb) a week
**Training period**	3 weeks	12 weeks	12 weeks

UC: uniform continuous; PC: progressive continuous; VC: varying continuous; RPM: laps for minute.

## Data Availability

No new data were created or analyzed in this study. Data sharing is not applicable to this article.
